# Predictors of poor outcome after both column acetabular fractures: a 30-year retrospective cohort study

**DOI:** 10.1186/1754-9493-7-9

**Published:** 2013-03-19

**Authors:** Philipp Lichte, Richard M Sellei, Philipp Kobbe, Derek G Dombroski, Axel Gänsslen, Hans-Christoph Pape

**Affiliations:** 1 Department of Orthopaedic Trauma Surgery, Medical School of the RWTH Aachen, Aachen, Germany; 2 Department of Trauma Surgery, Klinikum Wolfsburg, Wolfsburg, Germany; 3 Department of Orthopaedic Surgery, Parkland Memorial Hospital, Dallas, TX, USA

**Keywords:** Both column acetabular fractures, Outcome prediction, Long term results

## Abstract

**Background and Purpose:**

Acetabular fractures are often combined with associated injuries to the hip joint. Some of these associated injuries seem to be responsible for poor long-term results and these injuries seem to affect the outcome independent of the quality of the acetabular reduction. The aim of our study was to analyze the outcome of both column acetabular fractures and the influence of osseous cofactors such as initial fracture displacement, hip dislocation, femoral head lesions and injuries of the acetabular joint surface.

**Methods:**

A retrospective cohort study in patients with both column acetabular fractures treated over a 30 year period was performed. Patients with a follow-up of more than two years were invited for a clinical and radiological examination. Displacement was analyzed on initial and postoperative radiographs. Contusion and impaction of the femoral head was grouped. Injuries of the acetabular joint surface consisting of impaction, contusion and comminution were recorded. The Merle d’Aubigné Score was documented and radiographs were analysed for arthritis (Helfet classification), femoral head avascular necrosis (Ficat/Arlet classification) and heterotopic ossifications (Brooker classification).

**Results:**

115 patients were included in the follow up examination. Anatomic reduction (malreduction ≤ 1mm) was associated with a significantly better clinical outcome than nonanatomical reduction (p = 0.001). Initial displacement of more than 10mm (p = 0.031) and initial intraarticular fragments (p = 0.041) were associated with worse outcome. Other associated injuries, such as the presence of a femoral head dislocation, femoral head injuries and injuries to the acetabular joint surface showed no significant difference in outcome individually, but in fractures with more than two associated local injuries the risk for joint degeneration was significant higher (p < 0.001) than in cases with less than two of them.

In the subgroup of anatomically reconstructed fractures no significant influence of the analyzed cofactors could be observed.

**Conclusion:**

Anatomical reduction appears to be an important parameter for a good clinical outcome in patients with both column acetabular fractures. Additional fracture characteristics such as the initial displacement and intraarticular fragments seem to influence the results. Patients should also be advised that both column acetabular fractures with more than two additional associated factors have a significantly higher risk of joint degeneration.

## Background

Long-term outcome is a major concern for patient communication when discussing the therapy. Especially in acetabular fractures, an adverse outcome is often associated with immobilization and the need for arthroplasty.

Numerous follow-up studies have been performed in acetabular injuries [1-4].

However, most of these studies have summarized the results of different types of acetabular fractures. Overall, satisfactory results occurred in 60-85% of the cases [2,3,5,6] but almost all of these describe the type of acetabular fracture and the relevant impact on the long term result [6,7]. Fractures of the anterior column or isolated transverse fractures appear to show better results than the combination of transverse - posterior wall fractures [1,3]. In general, the accuracy of reduction plays an important role in the outcome. This surgeon-related factor is especially true for the posterior wall fractures. However, information on both column acetabular fractures is limited [6].

Besides the fracture type and the quality of reduction some associated injuries may influence the long-term outcome. In particular lesions of the femoral head [1,8], acetabular comminution [9,10] and intraarticular fragments [11] seem to impair the results. The relevance of these injuries is not yet sufficiently described for both-column acetabular fractures.

The goal of our study is to address the following questions:

1. Does the accuracy of anatomic reduction play a role in the risk of arthritis in both column acetabular fractures?

2. Which individual injury-associated factors or a combination can be used to predict the outcome before advising the patient?

## Methods

We performed a follow up study in patients from a database where all treated patients with acetabular fractures were prospectively included over a 30-year period (January 1 1974 until the end of 2003). Inclusion criteria for our study were the presence of a both column acetabular fracture an age of at least sixteen and complete documentation of the below mentioned parameters.

The following parameters were recorded from the database: age, sex, mechanism, injury pattern, injury severity (according to the Injury Severity Score, ISS), concomitant injuries, diagnostics, therapy, in-patient course, hip dislocation and time to reduction, fracture displacement, femoral head injuries, injuries of the acetabular joint surface, presence of intraarticular fragments and death. The quality of reduction was classified according to Matta’s criteria [5] on conventional AP pelvic, iliac and obturator oblique, inlet and outlet X-rays.

### Follow up examination

The clinical follow-up was performed at least 2 years after injury referring to previous studies [12,13]. Follow-up examinations were all performed by the same observer based on a standardized questionnaire and a standardized documentation sheet. In addition to the physical examination, plain x-rays of the pelvis were taken.

We documented the Merle-d’Aubigné Score (MAS) [14,15], the Brooker classification of heterotopic ossification [16], the Helfet classification of posttraumatic arthritis and the Ficat and Arlet classification of avascular necrosis of the femoral head [17].

Radiological joint degeneration was defined as:

•Grade 3 or 4 posttraumatic arthritis (Helfet)

•Stage 3 or 4 femoral head necrosis (Ficat/Arlet)

•Grade III or IV heterotopic ossification (Brooker)

•or total hip arthroplasty.

We also analyzed the influence of a number of associated injuries (hip dislocation, intraarticular fragments, initial displacement >10mm, femoral head or acetabular joint surface impaction/bruise) on the radiological outcome.

### Statistics

The association between clinical and radiological outcome was determined by Pearson Chi-Square-test. To assess the influence of categorical parameters on the outcome, the Pearson Chi-Square-test and the Fisher-Yates-test were used. To analyze the influence of continuous numeric parameters on the outcome the Mann–Whitney Test was used. For all tests a significant correlation was assumed if the p value was <0.05. The study protocol was approved by the Institutional Review Board and the Ethics committee of Hannover Medical School. All of the participants provided written informed consent.

## Results

Two hundred six patients with both column acetabular fractures were treated. One hundred fifteen of the 206 patients (54.9%) participated in the follow up examination. Twenty six of the excluded patients died in hospital due to direct consequences of the accident. Twenty one died due to other reasons; 29 were not reachable. Two were wheelchair bound due to amputation of the upper leg, and 13 refused the participation.

The mean time between the injury and follow-up examination was 5.2 years (2–19 years).

Demographic data of the included patients are shown in Table 1.

**Table 1:** Demographic data

	
Included patients	115
Mean age in years (range)	40 (16–89)
Male:female (%)	80:35 (69.6:30.4)
Mean ISS (range)	18.6 (6–66)
ISS >16 (%)	53 (46.1)

Almost 60% of the patients (59.7%) suffered a high-energy trauma (Table 2). Ninety eight percent of the patients had injuries in other body regions. In most of these patients (51; 46.8%) a traumatic brain injury was diagnosed, followed by thoracic (41; 37.6%) and abdominal injuries (21; 19.3%).

**Table 2:** Main causes of the injury

	
Car accident (%)	57 (52.3)
Fall from great height	11 (10.1)
Fall over	11 (10.1)

### Treatment

Of all the patients available for follow up, 71.3% (n = 82) of the fractures were treated operatively. Indications for non-operative treatment were:

•Nondisplaced fractures or fractures with minimal displacement (< 2mm)

•and absence of hip dislocation, femoral head injuries, comminution and intraarticular fragments.

For operative treatment several approaches were used according to the fracture morphology (50 were anterior, 11 posterior and 21 combined approaches).

### Outcome

Clinical outcome measurements revealed that according to the Merle-d’Aubigné-score 40% (n = 46) of the patients showed no measurable functional limitations of the hip. 34.8% (n = 40) of the patients showed slight (15–17 points) and 6.1% (n = 7) moderate limitations (13–14 points). Severe limitations of function (<13 points) were observed in 19.1% (n = 22). The average value of the MAS was 15.7 points (6–18 points).

The results of radiological follow up are shown in Table 3. Altogether 23 patients (20%) were determined to have radiological joint degeneration (n = 12) or underwent a total hip replacement (n = 11).

**Table 3:** Results of radiological follow up (collective group)

	
Helfet grade 1	60.0% (n = 69)
Helfet grade 2	17.4% (n = 20)
Helfet grade 3	6.1% (n = 7)
Helfet grade 4	16.5% (n = 19)
Ficat/Arlet stadium 0	95.7% (n = 110)
Ficat/Arlet stadium 1	1.1% (n = 1)
Ficat/Arlet stadium 2	(n = 0)
Ficat/Arlet stadium 3	(n = 0)
Ficat/Arlet stadium 4	3.2% (n = 3)
Brooker 0	53.0% (n = 61)
Brooker I	5.2% (n = 6)
Brooker II	16.5% (n = 19)
Brooker III	16.5% (n = 19)
Brooker IV	8.7% (n = 10)

**Figure 1:**
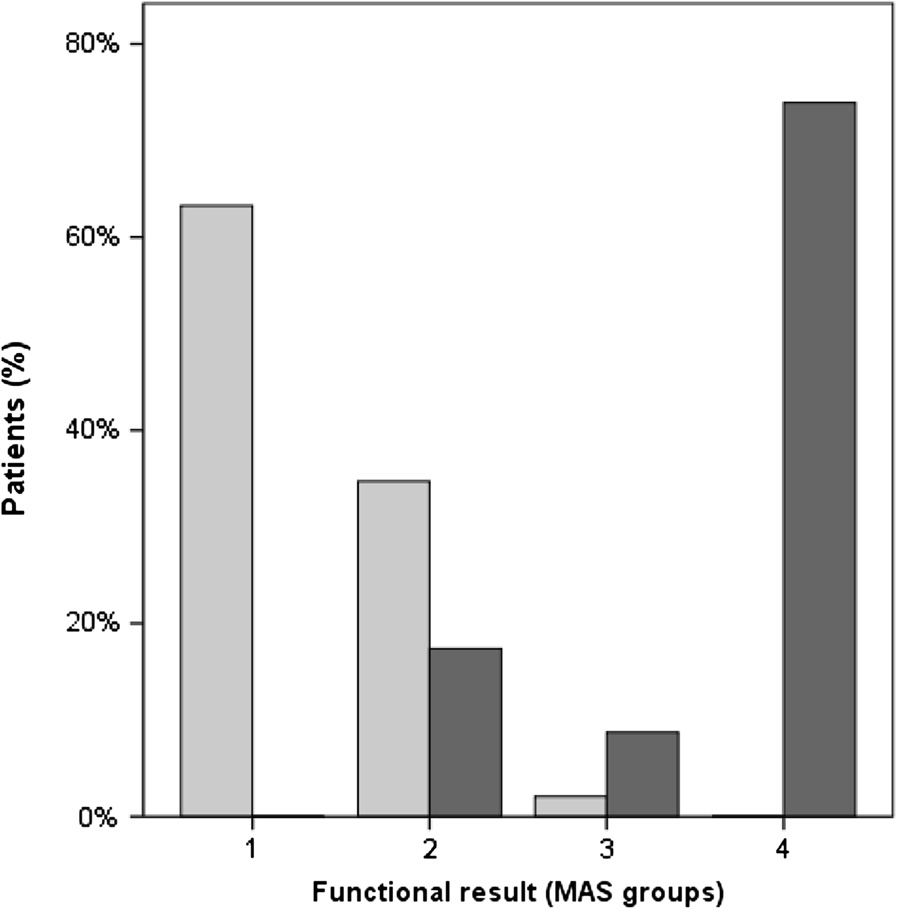
**Correlation between radiological outcome and MAS.** The percentage share of patients with good radiological outcome is shown with the light grey columns. The percentage share of patients with radiological joint degeneration (Helfet, Brooker, Ficat/Arlet stadium 3 and 4) is shown with the dark grey columns. MAS groups were defined as group 1 (no functional limitations (18 points), group 2 (slight limitations (15–17 points)), group 3 (moderate limitations (13–14 points)) and group 4 (severe limitations (<13 points)).

Comparing the clinical and radiological results we observed an association between the Merle-d’Aubigné-Score and the radiological results (Figure 1). The average MAS for patients with good radiological results was 17.5 (14–18), and patients with poor radiological results had an average MAS of 12.0 (7–17) points (p = 0.001).

There were no significant differences in the mean ISS between patients with good vs. poor results.

### Influence of reduction on the outcome

The average value of the MAS was significantly higher in the group with anatomical reduction (reduction <1mm) than in the group with imperfect reduction (malreduction ≥1mm) (17.5 vs. 12, p = 0.001).

### Influence of associated cofactors on the outcome

The comparison of the average initial displacement of fractures with good and poor radiological results shows a highly significant difference (11.4mm (0-39mm) vs. 17.8mm (8-37mm), p = 0.008). The rate of joint degeneration was significantly higher if the primary displacement was more than 10mm (10.9% (5/46) vs. 27.5% (19/69), p = 0.031).

Intraarticular fragments were associated with a significant increase of radiological joint degeneration (50% (4/8) vs. 18% (18/95), p = 0.041).

Bone bruise, impaction of the femoral head (p = 0.599) and bruise or impaction of the acetabular joint surface (p = 0.611) were not significantly associated with a worse radiological outcome. Patients with a fracture-dislocation of the hip also showed no significant increase in joint failure (p = 0.912).

**Figure 2:**
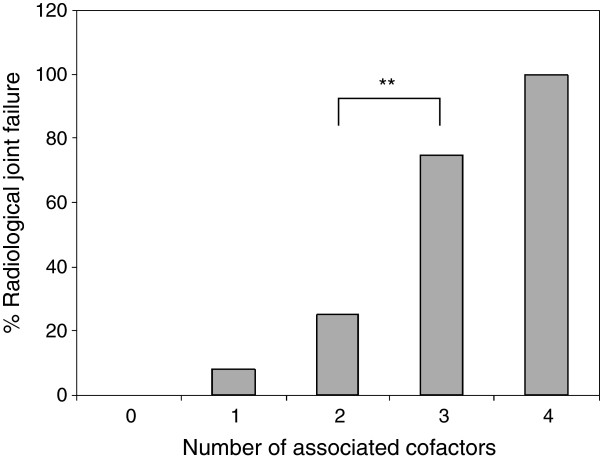
**The percentage of radiological joint failure increases in case of increasing number of associated cofactors (hip dislocation, intraarticular fragments, femoral head or acetabular joint surface impaction, initial displacement >10mm).** ** p = 0.001.

Additionally, we analysed how more than two associated local cofactors influenced the long term results. We found a highly significant (p < 0.001) increased risk for joint degeneration in these cases when compared with those that had two or fewer associated cofactors (Figure 2).

### Subgroup analysis of cases with anatomical reduction

To assess whether additional factors can influence the outcome of both column acetabular fractures independent of the reduction quality, we analyzed the subgroup of surgically treated patients with anatomical reconstruction of the acetabular joint (n = 54).

Results of radiological follow up are shown in Table 4. In summary, 50.0% (n = 27) of the fractures had a good radiological result (Helfet I/II, Ficat/Arlet I/II, Brooker I/II). Radiological joint degeneration was observed in 20.4% (n = 11) of patients, and 11.1% (n = 6) received a total hip replacement.

**Table 4:** Results of radiological follow up (subgroup analysis of patients after anatomical reduction)

	
Helfet grade 1	44.4% (n = 24)
Helfet grade 2	14.8% (n = 8)
Helfet grade 3	20.4% (n = 11)
Helfet grade 4	20.4% (n = 11)
Ficat/Arlet stadium 0	81.5% (n = 44)
Ficat/Arlet stadium 1	7.4% (n = 4)
Ficat/Arlet stadium 2	3.7% (n = 2)
Ficat/Arlet stadium 3	5.6% (n = 3)
Ficat/Arlet stadium 4	1.9% (n = 1)
Brooker 0	51.9% (n = 28)
Brooker I	14.8% (n = 8)
Brooker II	20.4% (n = 11)
Brooker III	9.3% (n = 5)
Brooker IV	3.7% (n = 2)

We could not find a significantly higher rate of radiological joint failure in patients with a dislocated hip (p = 0.45), additional injuries of the femoral head (p = 0.38), acetabular comminution (p = 0.741) or acetabular bruise-/impaction (p = 0.067).

## Discussion

The long-term prognosis of both column acetabular fractures corresponds to the results of many previous studies of acetabular fractures which reported good or very good functional results in 60-85% of the cases [18,19]. In Matta’s study the number of surgically treated both-column fractures was 18.6% (n = 92). Seventy seven percent of these presented with excellent or good long-term clinical results [1]. Mayo showed good or better results for 75% (n = 124) of both column fractures, and in other studies, 88% of these fractures had at least good outcomes [3,20].

Our main results are as follows:

1. 74.1% of the patients demonstrated a good clinical outcome.

2. The clinical result (MAS) of the fractures with anatomical reductions (79.5%) was significantly better than those with nonanatomical reductions.

3. The primary displacement of the fragments and the presence of intraarticular fragments are relevant for the outcome.

4. Overall the presence of more than two associated local cofactors also correlates with poor outcome.

**Figure 3:**
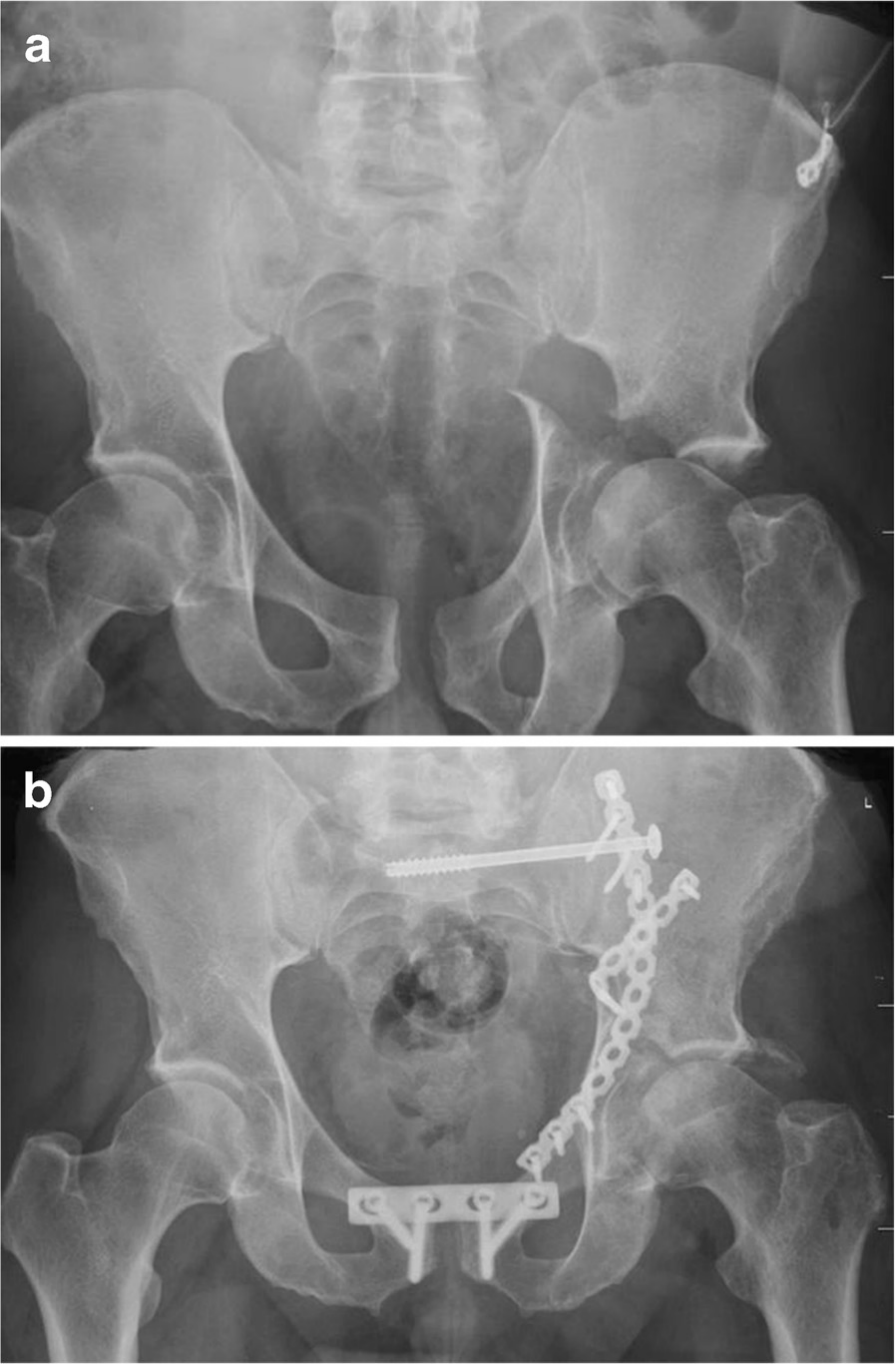
**a) This is a x-ray of a 48 year old motor cyclist, who suffered an accident with a both column acetabular fracture accompanied by a pelvic ring injury.** Due to a massive soft tissue involvement of the pelvis and prolonged wound healing, an isolated ilio-inguinal approach was applied to reduce and stabilize the fracture. Therefore a nonanatomical reduction had to be accepted. **b**) X-ray 4 months after surgery. The combination of great initial displacement and nonanatomical reduction led to a rapid joint degeneration with poor clinical function.

The significant influence of anatomic reconstruction, *e.g*. the congruence of the femoral head and the acetabular joint surface after fracture healing has been validated in several studies [1,5,21-27]. Letournel showed a portion of 22.7% both-column fractures. Postoperatively excellent or good results of reduction were observed in 73%. Eighty two percent of these patients presented excellent or good long term results [6]. Ovre et al. found a high correlation between non-anatomical reduction in surgically treated acetabular fractures and two year outcome [28]. Isolated posterior wall fractures have a strong correlation between the quality of reduction and measured outcomes [9,10,24,29,30]. Matta reports that even after apparently anatomical reduction some residual surface irregularities can remain. These may be responsible for changes in pressure distribution in the hip joint and require the hip joint to compensate by remodelling [1]. Kreder et al. concluded that anatomical reduction alone might not be sufficient for good functional results after posterior wall fractures and that the fracture pattern and little residual displacement are responsible for the development of arthritis [31]. Nonetheless, our data suggests that anatomical reduction of both column acetabular fractures is an important factor to achieve good clinical results (Figure 3a and b) but does not guarantee a good outcome.

The question of which factors determine poor outcomes is not yet conclusively clarified and may be complicated by the differences in the fractures types. Murphy et al. described four additional pathologies (associated fracture type, imperfect reduction with malreduction >3mm, presence of local complications and heterotopic bone), which he used as prognostic factors in outcome of all acetabular fractures [32]. Also traumatic lesions of the acetabular articular surface have been identified to have a negative impact on the outcome [33]. Other studies showed controversial results concerning the influence of these different factors. Our study is in keeping with those previous reports in that cofactors are relevant for the outcome after acetabular fractures.

While some individual cofactors had no significant effect on outcome, our data show that increasing the number of these additional local injuries correlates with worse results. This correlation indicates that the severity of additional local injuries should not be underestimated. This caveat is supported by our finding that all isolated both column acetabular fractures with no associated cofactors had good outcomes. Moreover patients suffering more than two associated local injuries have a significantly higher risk of joint degeneration. What is interesting is that subgroup analysis of anatomically reduced fractures showed these factors no longer negatively influenced outcome.

In our study the non-operative and operative treatment group showed similar results. This may be a hint that non-operative treatment may be a save option in patients with a fracture displacement <2mm. Due to the study design we are not able to give a strong recommendation for treatment strategies.

### Strength and weaknesses of the study

We feel that the number of patients followed is a strength because it is at least in line with the amount of both column fractures in other studies [1,4].

Also, the mean follow up of over 5 years provides a good overview on complication rates and arthritis [34,35] and is comparable to other current acetabular follow up studies [33,36].

Follow up examinations and x-ray classifications were personally performed by one investigator to avoid an interobserver variability. Nevertheless inconsistencies in quantifying the cofactors especially impaction and contusion cannot be excluded.

The high number of treating surgeons is related to the long period of data collection. Due to this variable and a lacking standardized treatment protocol we were not able to analyse correlations between treatment procedures and results.

## Conclusion

We conclude that associated local injuries have an impact on the result and should receive attention when advising the patient about long term results. Anatomical reduction is the main factor for good outcome in both column acetabular fractures whereas greater initial displacement and intraarticular fragments are predictors for a worth result. Patients should be informed that the more associated cofactors present the worse the outcome prognosis will be.

## Competing interests

The authors declare that they have no competing interests.

## Authors’ contribution

PL and HCP conceived and drafted the manuscript. HCP and AG had managed the registry and performed the follow up examinations. PL, PK, RS and DGD were involved in statistical analysis and data evaluation. All authors participated in the critical revision of the manuscript. All authors read and approved the final manuscript.
